# Supplementary material for: Causal Relationship between Aging and Anorexia Nervosa: A White-Matter-Microstructure-Mediated Mendelian Randomization Analysis
Source: Biomedicines. 2024 Aug 16;12(8):1874. doi: 10.3390/biomedicines12081874 (PMC11351342; doi:10.3390/biomedicines12081874)
Supplement: Supplementary file 1 [file biomedicines-12-01874-s001.zip › Supplementary Figure/Figure S4/Singe SNP plot of MR analysis for TL to ubm-b-1788.pdf]

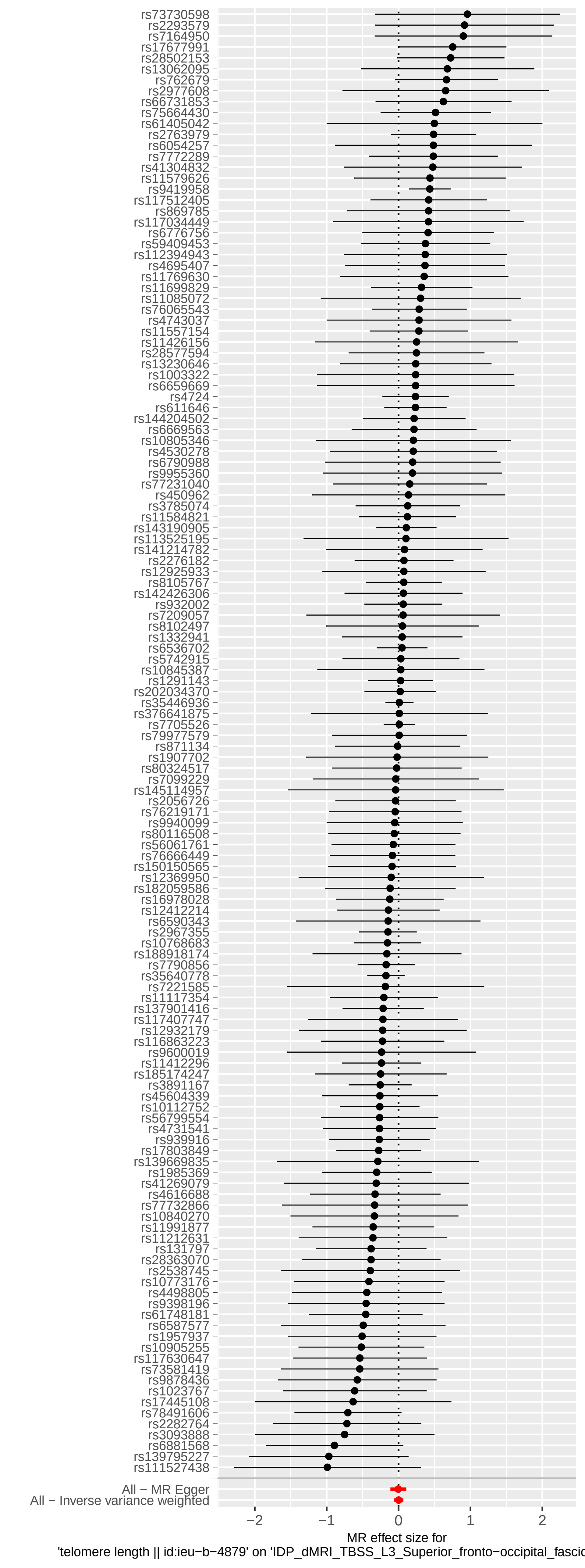
rs73730598
rs2293579
rs7164950
rs17677991
rs28502153
rs13062095
rs762679
rs2977608
rs66731853
rs75664430
rs61405042
rs2763979
rs6054257
rs7772289
rs41304832
rs11579626
rs9419958
rs117512405
rs869785
rs117034449
rs6776756
rs59409453
rs112394943
rs4695407
rs11769630
rs11699829
rs11085072
rs76065543
rs4743037
rs11557154
rs11426156
rs28577594
rs13230646
rs1003322
rs6659669
rs4724
rs611646
rs144204502
rs6669563
rs10805346
rs4530278
rs6790988
rs9955360
rs77231040
rs450962
rs3785074
rs11584821
rs143190905
rs113525195
rs141214782
rs2276182
rs12925933
rs8105767
rs142426306
rs932002
rs7209057
rs8102497
rs1332941
rs6536702
rs5742915
rs10845387
rs1291143
rs202034370
rs35446936
rs376641875
rs7705526
rs79977579
rs871134
rs1907702
rs80324517
rs7099229
rs145114957
rs2056726
rs76219171
rs9940099
rs80116508
rs56061761
rs76666449
rs150150565
rs12369950
rs182059586
rs16978028
rs12412214
rs6590343
rs2967355
rs10768683
rs188918174
rs7790856
rs35640778
rs7221585
rs11117354
rs137901416
rs117407747
rs12932179
rs116863223
rs9600019
rs11412296
rs185174247
rs3891167
rs45604339
rs10112752
rs56799554
rs4731541
rs939916
rs17803849
rs139669835
rs1985369
rs41269079
rs4616688
rs77732866
rs10840270
rs11991877
rs11212631
rs131797
rs28363070
rs2538745
rs10773176
rs4498805
rs9398196
rs61748181
rs6587577
rs1957937
rs10905255
rs117630647
rs73581419
rs9878436
rs1023767
rs17445108
rs78491606
rs2282764
rs3093888
rs6881568
rs139795227
rs111527438
All − MR Egger
All − Inverse variance weighted
−2
−1
0
1
2
MR effect size for
'telomere length || id:ieu-b-4879' on 'IDP_dMRI_TBSS_L3_Superior_fronto−occipital_fascic
